# The lncRNA PVT1 regulates nasopharyngeal carcinoma cell proliferation via activating the KAT2A acetyltransferase and stabilizing HIF-1α

**DOI:** 10.1038/s41418-019-0381-y

**Published:** 2019-07-18

**Authors:** Ying Wang, Wanyuan Chen, Jiayan Lian, Haibo Zhang, Bo Yu, Minjun Zhang, Fangqiang Wei, Jianhui Wu, Jiaxiang Jiang, Yongshi Jia, Fan Mo, Shirong zhang, Xiaodong Liang, Xiaozhou Mou, Jianming Tang

**Affiliations:** 1 Department of Radiation Oncology, Zhejiang Provincial People’ s Hospital, People’ s Hospital of Hangzhou Medical College, Hangzhou, 310014 Zhejiang PR China; 2 Department of Pathology, Zhejiang Provincial People’s Hospital, People’s Hospital of Hangzhou Medical College, Hangzhou, 310014 Zhejiang PR China; 3 Department of Pathology, the 7th affiliated hospital of Sun Yat-Sen University, Shenzhen, 510275 Guandong PR China; 4 State Key Laboratory of Oncogenes and Related Genes, Renji-Med X Clinical Stem Cell Research Center, Ren Ji Hospital, School of Medicine, Shanghai Jiao Tong University, 200127 Shanghai, PR China; 5 Graduate Department, Bengbu Medical College, Bengbu, 233000 Anhui PR China; 6 Department of Hepatobiliary and Pancreatic Surgery, Zhejiang Provincial People’s Hospital, People’s Hospital of Hangzhou Medical College, Hangzhou, 310014 Zhejiang PR China; 7 Department of The Otolaryngology, Zhongshan City People’s Hospital, Zhongshan Affiliated Hospital of Sun Yan-sen University, Zhongshan, 528403 Guangdong PR China; 8 Department of Cardiology, Zhejiang Provincial People’s Hospital, People’s Hospital of Hangzhou Medical College, Hangzhou, 310014 Zhejiang PR China; 9 Vancouver Prostate Centre, University of British Columbia, 2660 Oak St., Vancouver, BC V6H 3Z6 Canada; 10 Translational medicine centre, Affiliated Hangzhou First People’s Hospital, Zhejiang University School of Medicine, Hangzhou, 310006 Zhejiang PR China; 11 Key Laboratory of Tumor Molecular Diagnosis and Individualized Medicine of Zhejiang Province, Zhejiang Provincial People’s Hospital, People’s Hospital of Hangzhou Medical College, Hangzhou, 310014 Zhejiang PR China; 12 Clinical Research Institute, Zhejiang Provincial People’s Hospital, People’s Hospital of Hangzhou Medical College, Hangzhou, 310014 Zhejiang PR China

**Keywords:** Oncogenes, Protein folding

## Abstract

Long noncoding RNAs (lncRNAs) play important roles in regulating the development and progression of many cancers. However, the clinical significance of specific lncRNAs in the context of nasopharyngeal carcinoma (NPC) and the molecular mechanisms by which they regulate this form of cancer remain largely unclear. In this study we found that the lncRNA PVT1 was upregulated in NPC, and that in patients this upregulation was associated with reduced survival. RNA sequencing revealed that PVT1 was responsible for regulating NPC cell proliferation and for controlling a hypoxia-related phenotype in these cells. PVT1 knockdown reduced NPC cell proliferation, colony formation, and tumorigenesis in a subcutaneous mouse xenograft model systems. We further found that PVT1 serves as a scaffold for the chromatin modification factor KAT2A, which mediates histone 3 lysine 9 acetylation (H3K9), recruiting the nuclear receptor binding protein TIF1β to activate NF90 transcription, thereby increasing HIF-1α stability and promoting a malignant phenotype in NPC cells. Overexpression of NF90 or HIF-1α restored the proliferation in cells that had ceased proliferating due to PVT1 or KAT2A depletion. Conversely, overexpression of active KAT2A or TIF1β, but not of KAT2A acetyltransferase activity-deficient mutants or TIF1β isoforms lacking H3K9ac binding sites, promoted a PVT1-mediated increase in NF90 transcription, as well as increased HIF-1α stability and cell proliferation. PVT1 knockdown enhanced the radiosensitization effect in NPC cells via inhibiting binding between H3K9ac and TIF1β in a manner. Taken together, our results demonstrate that PVT1 serves an oncogenic role and plays an important role in radiosensitivity in malignant NPC via activating the KAT2A acetyltransferase and stabilizing HIF-1α.

## Introduction

Nasopharyngeal carcinoma (NPC), which is a form of cancer arising from the epithelium of the nasopharynx, remains highly prevalent, particularly in Southeast Asia and Southern China [1, 2]. Although intensity-modulated radiation advances in NPC treatment, tumor proliferation, and growth for NPC patients remain to be the important cause of treatment failure and cancer-related death [3, 4]. Recently, an increasing body of evidence has suggested that long noncoding RNAs (lncRNAs) are involved in tumorigenesis through regulating histone modification [5, 6]. However, the mechanisms that account for it remain to be elucidated.

Plasmacytoma variant translocation 1 (PVT1) is a lncRNA that has been found to serve an oncogenic role in a variety of malignant tumors. PVT1 was first discovered to be frequently translocated in mouse models of plasmacytoma, ultimately contributing to carcinogenesis in these models [7, 8]. Recent evidence further indicates that PVT1 exhibits aberrant expression in nonsmall-cell lung cancer [9–11], cervical cancer [10], colorectal cancer [12], and gastric cancer [13, 14]. Moreover, PVT1 expression is significantly linked to patient survival in those with colorectal [15], lung [16], and breast cancer [17]. PVT1 has been shown to directly bind and stabilize the KLF5 proteins in breast cancer [17]. Enhancer of zeste homolog 2 (EZH2), a major histone methyltransferase, plays an essential role in tumor regulation via trimethylating lysine 27 on histone H3. EZH2 forms a molecular complex with PVT1 to function as an repressive driver of p15 and p16 in gastric cancer [18]. PVT1 is also transcriptionally activated by FOXM1 in gastric cancer [19]. Furthermore, PVT1 induces radioresistance by influencing cell apoptosis and DNA repair in NPC [20]. However, the specific biological importance and clinical significance of PVT1 in NPC progression remains to be established.

In the present study, we found that PVT1 was upregulated in NPC, and that it predicted poor survival in patients. PVT1 promoted this NPC cell proliferation via activating the KAT2A H3K9 acetyltransferase and TIF1β activity to activate NF90 transcription and increase HIF-1α stability. Interestingly, PVT1 contributed to the radiosensitization effect in NPC cells by enhancing the binding of H3K9ac and TIF1β in a manner. Collectively, our results establish a new regulatory mechanism by which PVT1 promotes NPC progression, providing a potential therapeutic target and prognostic factor for NPC.

## Results

### The PVT1 lncRNA is upregulated in NPC and is associated with a poor prognosis in patients

**Fig. 1:**
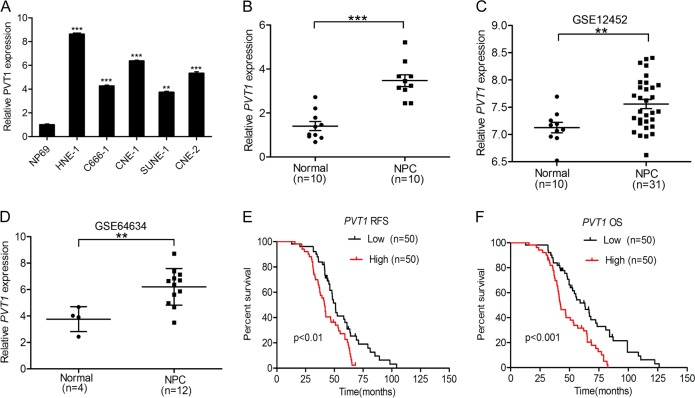
The PVT1 lncRNA is upregulated in NPC and is associated with a poor prognosis in patients. **a** Expression of PVT1 mRNA is higher in NPC cell lines compared with the immortalized nasopharyngeal epithelial cell line. **b** Expression levels of PVT1 mRNA in ten clinical NPC tumors and ten freshly frozen normal nasopharyngeal specimens. **c**, **d** Expression levels of PVT1 mRNA in NPC tumors and normal nasopharyngeal specimens. PVT1 mRNA-expression data were obtained from the GSE12452 dataset [21] and the GSE64634 dataset [22] and analyzed. **e**, **f**, Kaplan–Meier analysis of relapse-free survival (RFS) and over survival (OS) for patients with high PVT1 mRNA-expression NPC versus low PVT1 mRNA-expression NPC. Error bars ± SD. **P < 0.01. ***P < 0.001. Data are representative from three independent experiments

To identify the functions of PVT1 in NPC progression, we first analyzed PVT1 expression in the NP69 immortalized nasopharyngeal epithelial cell line and in five NPC cell lines (HNE-1, C666-1, CNE-1, SUNE-1, and CNE-2). Interestingly, we found that the PVT1 expression level was higher in NPC cell lines than that in NP69 cells (Fig. 1a). We next examined PVT1 expression in ten freshly frozen normal nasopharyngeal specimens and in ten clinical NPC tumor samples. As shown in Fig. 1b, compared with the normal nasopharyngeal epithelial specimens, PVT1 expression was markedly elevated in NPC tumors. To further confirm this finding, we obtained gene expression data from the microarray datasets GSE12452 [21] and GSE64634 [22], and examined PVT1 was more highly expressed in NPC tissues relative to normal nasopharyngeal tissues (Fig. 1c, d). These results suggest that PVT1 may function as an oncogene that is involved in NPC progression.

Next, to assess clinical relevance of PVT1 expression in NPC, we examined the relationship between PVT1 expression and NPC patient survival via a Kaplan–Meier survival analysis. As shown in Fig. 1e, f, patients who exhibited high PVT1 expression had a shorter relapse-free survival and over survival. This indicates that PVT1 is closely associated with a poor prognosis in NPC patients. Moreover, we demonstrated that PVT1 is abundant within both the nucleus and cytoplasm of NPC (Supplementary Fig. 1A, B). Taken together, these results indicate that PVT1 is upregulated in NPC and is associated with a poor prognosis in NPC patients.

### Knockdown of PVT1 disrupts NPC cell proliferation and tumor growth

**Fig. 2:**
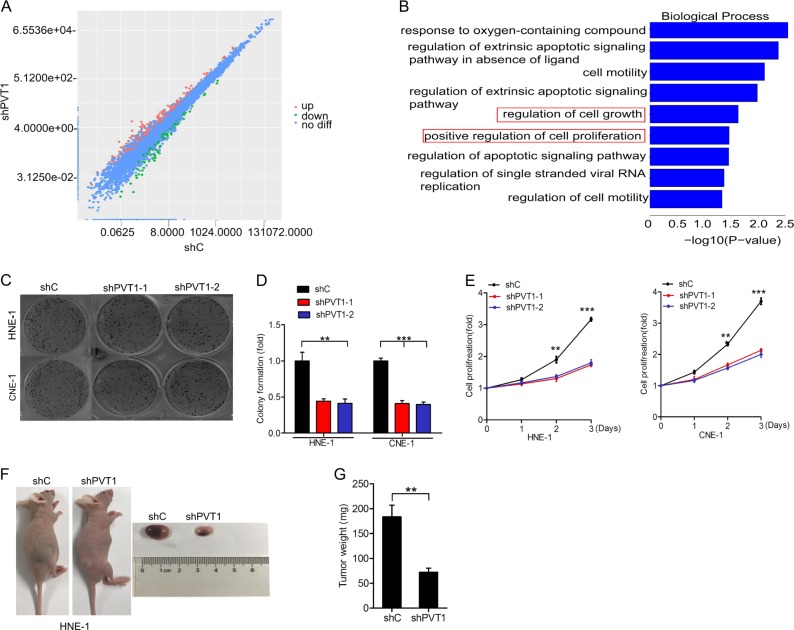
Knockdown of PVT1 disrupts NPC cell proliferation and tumor growth. **a** Scatter plot of 16764 gene expression in HNE-1 cells transfected with PVT1 shRNA (*y*-axis) compared with the control group (*x*-axis). Green dots, set of genes exhibiting significant downregulation upon PVT1 knockdown; red dots, set of genes exhibiting significant upregulation upon PVT1 knockdown; black dots, the genes without significant change. **b** The biological process signaling pathways response upon PVT1 knockdown by Gene GO analyses. **c** Effects of PVT1 knockdown on NPC cell colony formation. **d** Quantification of colony formation in **c**. **e** Effects of PVT1 knockdown on NPC cell proliferation. **f** Representative images of PVT1 knockdown-inhibited HNE-1 subcutaneous tumor generation. Tumors were harvested at 3–4 weeks after implantation. Data were from two independent experiments with five mice per group with similar results. **g** Quantification of tumor weight in **f**. Error bars ± SD. **P < 0.01. ***P < 0.001. Data are representative from three independent experiments

To determine the functions of PVT1 in NPC, we first constructed HNE-1 cells expressing a PVT1-specific shRNA (shPVT1) or a nonsilencing control shRNA (shC), and then performed RNA sequencing to compare their gene expression profiles. As shown in Fig. 2a, 313 genes were upregulated and 137 genes were downregulated (≥1.5-fold, *p* < 0.05) in HNE-1 cells in which PVT1 was knocked down.

A gene ontology (GO) analysis was then performed in order to investigate the pathways responsive to PVT1 knockdown. Our results indicated that these differentially expressed genes were enriched in genes linked with proliferation (Fig. 2b). These results suggest that PVT1 may be a vital regulatory lncRNA involved in regulating NPC cell proliferation.

To provide functional confirmation of the above findings, we next revealed that PVT1 knockdown (Supplementary Fig. 2) inhibits colony formation (Fig. 2c, d) and cell proliferation in HNE-1 and CNE-1 cells (Fig. 2e). We next further sought to elucidate the biological role of PVT1 in NPC tumorigenesis, and found that PVT1 knockdown significantly inhibited NPC tumor growth in mice in the PVT1 silenced group relative to the control group (Fig. 2f, g). These results thus confirm that PVT1 plays a role in regulating the proliferation and growth of NPC tumors.

### PVT1 promotes NPC cell proliferation via HIF-1α

**Fig. 3:**
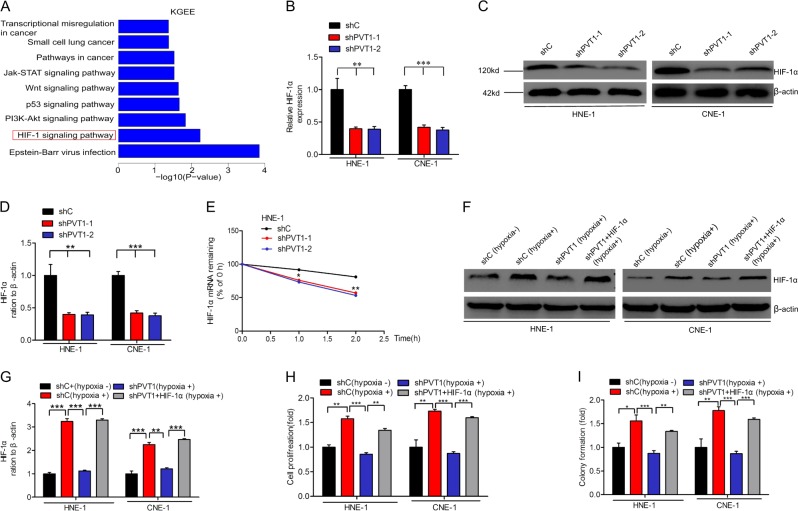
PVT1 promotes NPC cell proliferation via HIF-1α. **a** The signaling pathways response upon PVT1 knockdown by Gene KGEE analyses. Quantitative RT-PCR (**b**) and WB (**c**) analyses of effects of PVT1 knockdown on HIF-1α activation. **d** Quantification of HIF-1α protein in **c**. **e** Reduction of HIF-1α mRNA stability in PVT1 knockdown HNE-1 cells compared with control cells. Cells were treated with 1 μg/ml actinomycin D and RNA was extracted at the time of 0, 1, and 2 h. **f** HIF-1α protein expression assays for HNE-1 and CNE-1 under hypoxia conditions (1% O_2_). **g** Quantification of HIF-1α protein in **f**. Cell proliferation (**h**) and colony formation (**i**) assays for HNE-1 cells under hypoxic conditions. Hypoxia induced the cell proliferation and colony formation of NPC cells. The cell proliferation and colony formation abilities of NPC cells under hypoxic conditions were suppressed by PVT1 knockdown, and these suppressed cell proliferation and colony formation abilities of NPC cells were restored by HIF-1α overexpression. Error bars ± SD. *P < 0.05. **P < 0.01. ***P < 0.001. Data are representative from three independent experiments

To explore the mechanisms by which PVT1 regulates cell proliferation, we performed Kyoto Encyclopedia of Genes and Genomes (KGEE) to analyze the downstream pathways affected by PVT1 expression, revealing that PVT1 expression is positively correlated with the HIF-1 signaling pathway (Fig. 3a). Moreover, HIF-1α, which is a key mediator of the HIF-1 signaling pathway, mRNA (Fig. 3b) and protein levels (Fig. 3c, d) was downregulated upon PVT1 knockdown. These results suggest that HIF-1α may play a vital role in the PVT1 regulatory network.

Next, to determine whether PVT1 mediates HIF-1α mRNA stability, we treated HNE-1 cells with the RNA synthesis inhibitor Actinomycin D for 0, 1, or 2 h. Relative to the control group, we found that HIF-1α mRNA was degraded more quickly in the PVT1 depletion group (Fig. 3e), thus suggesting that PVT1 regulates HIF-1α mRNA stability.

To assess whether PVT1-mediated HIF-1α expression contributes to NPC cell proliferation, we overexpressed HIF-1α in PVT1-knockdown HNE-1 cells (Fig. 3f–g), and found that this restored the proliferation of these cells (Fig. 3h) and their colony formation abilities (Fig. 3i). These results further suggested that PVT1 thus regulates cell proliferation at least in part via modulating HIF-1α stability.

### PVT1 binds to KAT2A in NPC cells

**Fig. 4:**
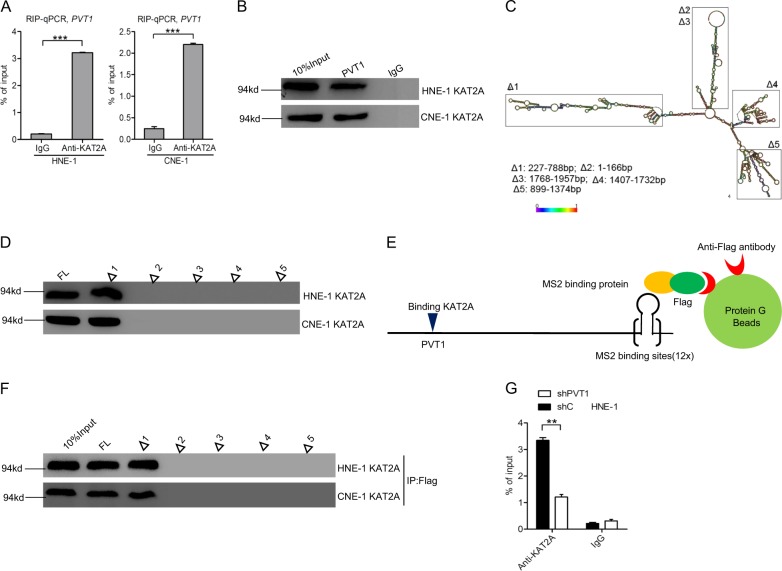
PVT1 binds to KAT2A in NPC cells. **a** The RIP-qPCR assay of the binding of KAT2A with PVT1 in HNE-1 and CNE-1 cells. **b** Biotinylated PVT1 was incubated with extracts (HNE-1 and CNE-1 cells), treated with streptavidin beads, and binding proteins were resolved in a gel. The WB assay of the specific binding of KAT2A and PVT1. **c** Graphic illustration of predicted PVT1 secondary structure (http://www.lncipedia.org), and the truncation of PVT1 in accordance with the stem-loop structure. **d** Secondary structure regions of PVT1 were treated as in **b**, and binding KAT2A was detected by the WB assay. **e**, **f**, WB detection of KAT2A binding upon secondary structure regions of PVT1 after Flag-MS2bp-MS2bs-based pull-down assay. **g** Effects of PVT1 knockdown on the specific binding of KAT2A and PVT1. Error bars ± SD. **P < 0.01. ***P < 0.001. Data are representative from two independent experiments

Recent studies have reported that lncRNAs can function as sponges to bind histone acetyltransferases and thereby modulate their downstream gene expression [5, 23]. We hypothesized that PVT1-mediated tumorigenesis may therefore depend upon its ability to bind certain histone acetyltransferases. We first found that three histone acetyltransferases (KAT2A, KAT2B, and KAT8) were markedly upregulated and two (KAT5 and KAT6A) were markedly downregulated in three freshly frozen normal nasopharyngeal specimens relative to three clinical NPC tumors via RNA sequencing (≥1.5-fold, *p* < 0.05) (Supplementary Fig. 3A). Next, we found that PVT1 bound to KAT2, but not to the other histone acetyltransferases tested (Fig. 4a and Supplementary Fig. 3B). Previous reports indicate that WDR5 and KAT2A can form a complex and bind to the lncRNA GClnc1 in order to promote gastric carcinogenesis [5]. Consistent with this mechanism, we found that the histone methyltransferase WDR5 was also clearly upregulated in NPC tissues relative to in normal nasopharyngeal tissues, and we further confirmed that PVT1 bound to WDR5 (Supplementary Fig. 3B). Moreover, we validated that both KAT2A and WDR5 protein expression levels were higher in NPC tissues compared with that in normal nasopharyngeal tissues (Supplementary Fig. 3C, D), but PVT1 knockdown had no effect on KAT2A or WDR5 expression (Supplementary Fig. 3E, F). These results thus suggest that PVT1 may act as a scaffold for the WDR5 and KAT2A complexes in order to specify a pattern of histone modification. We next utilized a biotin-avidin pull-down system to detect the association between PVT1 and KAT2A (Fig. 4b), and used deletion-mapping system (Fig. 4c, d) to detect whether KAT2A binds to with a specific region of PVT1. This approach identified a 227–788 nt region at the 5′ end of PVT1 required for its binding to KAT2A (Fig. 4d).

Finally, to further confirm that PVT1 binds to KAT2A, we used a Flag-MS2bp-MS2bs system to perform an RNA pull-down experiment (Fig. 4e). As shown in Fig. 4f, we found that KAT2A also bound to a 227–788 nt region at the 5′ end of PVT1 in HNE-1 cells. RIP quantitative real-time PCR demonstrated that PVT1 depletion inhibited the binding between PVT1 and KAT2A (Fig. 4g). Taken together, these results suggest that KAT2A binds to PVT1 in NPC cells.

### PVT1 stabilizes HIF-1α via KAT2A

**Fig. 5:**
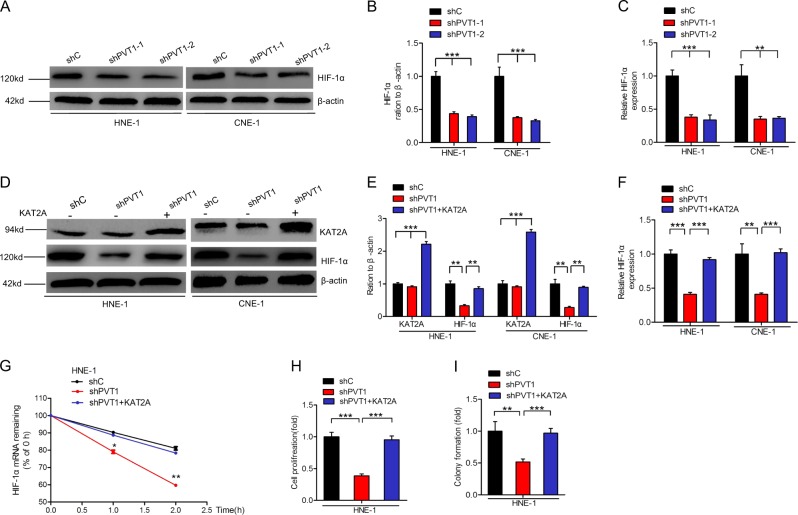
PVT1 stabilizes HIF-1α via KAT2A. **a** Effects of PVT1 knockdown on HIF-1α protein in HNE-1 and CNE-1 NPC cells. **b** Quantification of HIF-1α protein in **a**. **c** Effects of PVT1 knockdown on HIF-1α mRNA in HNE-1 and CNE-1 NPC cells. **d** Overexpression of KAT2A restores PVT1 knockdown-inhibited HIF-1α protein. **e** Quantification of HIF-1α and KAT2A proteins in **d**. Overexpression of KAT2A restores PVT1 knockdown-inhibited HIF-1α mRNA (**f**), HIF-1α mRNA stability (**g**), NPC cell proliferation (**h**), and colony formation (**i**). Error bars ± SD. **P < 0.01. ***P < 0.001. Data are representative from three independent experiments

As HIF-1α is a critical downstream effector of PVT1 in NPC cells, we next assessed whether PVT1 stabilizes HIF-1α via interacting with KAT2A in these cells. As shown in Fig. 5a–c, PVT1 depletion decreased HIF-1α protein and mRNA in HNE-1 and CNE-1 cells. However, overexpression of KAT2A rescued PVT1 knockdown-mediated loss of HIF-1α protein expression (Fig. 5d, e), mRNA expression (Fig. 5f), and mRNA stability (Fig. 5g). Moreover, overexpression of KAT2A restored PVT1 depletion-mediated loss of cell proliferation (Fig. 5h) and colony formation (Fig. 5i) in HNE cells. Taken together, these data show that the lncRNA PVT1 stabilizes HIF-1α via KAT2A.

### KAT2A acetyltransferase activity is required for PVT1-driven HIF-1α stabilization

**Fig. 6:**
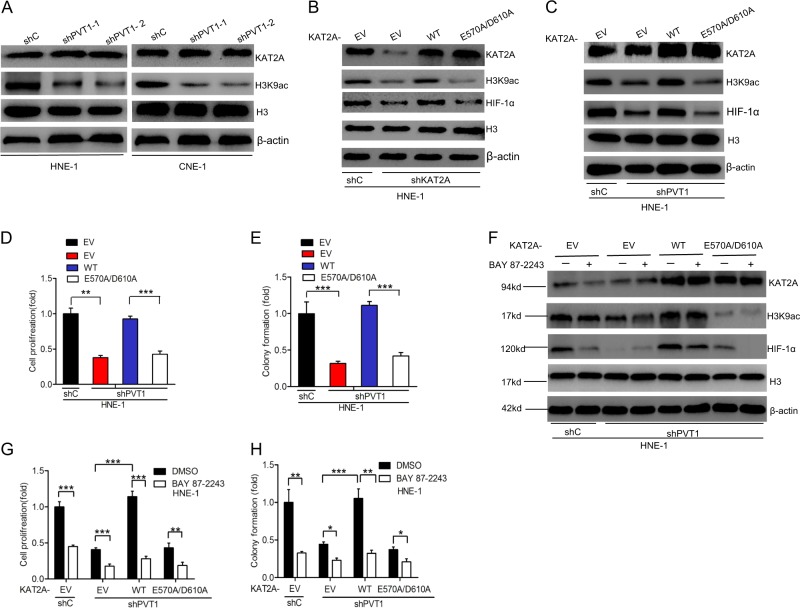
KAT2A acetyltransferase activity is required for PVT1-driven HIF-1α stabilization. **a** Effects of PVT1 knockdown on KAT2A and the acetylation of H3K9 expression in HNE-1 and CNE-1 NPC cells. β-actin and Histone H3 were used as controls. **b** Effects of overexpression of KAT2A wild-type and acetyltransferase activity-deficient mutant, E570A/D610A, on KAT2A depletion-inhibited HIF-1α expression. **c** Overexpression of KAT2A wild type but not acetyltransferase activity-deficient mutant, E570A/D610A, restored PVT1 depletion-inhibited HIF-1α expression. Effects of overexpression of KAT2A wild-type and acetyltransferase activity-deficient mutant, E570A/D610A, on NPC cell proliferation (**d**) and colony formation (**e**). Inhibition of HIF-1α reduced KAT2A-induced HIF-1α protein expression (**f**), NPC cell proliferation (**g**), and colony formation (**h**). Error bars ± SD. **P < 0.01, ***P < 0.001. Data are representative from three independent experiments

To explore the potential mechanisms by which PVT1 regulates HIF-1α stabilization through KAT2A, we next assessed histone H3 acetylation expression levels via western blotting. H3K9 has been reported to be a target of KAT2A [5]. As shown in Fig. 6a and Supplementary Fig. 4A, PVT1 knockdown decreased H3K9 acetylation levels, but not KAT2A expression in HNE-1 and CNE-1 cells. These data indicate that PVT1 may therefore mediate KAT2A acetyltransferase activity.

As the effects of PVT1-regulated KAT2A acetyltransferase activity in NPC cells are unclear, we focused on establishing the role of such acetyltransferase activity in the context of PVT1-regulated NPC cell proliferation. We therefore assessed whether KAT2A acetyltransferase activity was required for PVT1-regulated HIF-1α stabilization. As a recent study reported that KAT2A^E570A/D601A^ is an acetyltransferase activity-deficient mutant isoform of this protein [24], we constructed both KAT2A^WT^ and KAT2A^E570A/D601A^ plasmids. First, we examined that HIF-1α expression in KAT2A-knockdown HNE-1 cells is rescued by exogenous expression of KAT2A^WT^, but not by that of the KAT2A^E570A/D601A^ (Fig. 6b and Supplementary Fig. 4B). Moreover, as shown in Fig. 6c–e and Supplementary Fig. 4C, overexpression of KAT2A^WT^, but not KAT2A^E570A/D601A^, in HNE-1 NPC cells rescued PVT1 knockdown-mediated loss of HIF-1α expression, cell proliferation, and colony formation. This indicates that KAT2A acetyltransferase activity is important for PVT1-mediated HIF-1α stabilization.

Recent evidences have demonstrated that the lead structure BAY 87-2243 suppressed HIF-1α protein levels and reduced HIF-1 target gene expression [25, 26]. Then, we investigated whether treatment with the HIF-1α inhibitor BAY 87-2243 suppresses PVT1-mediated cell proliferation. As shown in Fig. 6f and Supplementary Fig. 4D, BAY 87-2243 treatment markedly suppressed HIF-1α expression in HNE-1 cells transfected the KAT2A WT relative to E570A/D601A Isoform or emptor vector (EV) controls, whereas H3K9ac levels were unaffected by BAY 87-2243 treatment. We next assessed the effects of BAY 87-2243 on PVT1-mediated cell proliferation and colony formation, and found that this inhibitor markedly disrupted NPC cell proliferation and colony formation in KAT2A^WT^, but not KAT2A^E570A/D601A^, overexpressing NPC cells relative to EV controls (Fig. 6g, h). These data demonstrate that KAT2A acetyltransferase activity is therefore required for PVT1-driven HIF-1α stabilization.

### PVT1 promotes HIF-1α stability via KAT2A-mediated regulation of the TIF1β/H3K9ac complex

**Fig. 7:**
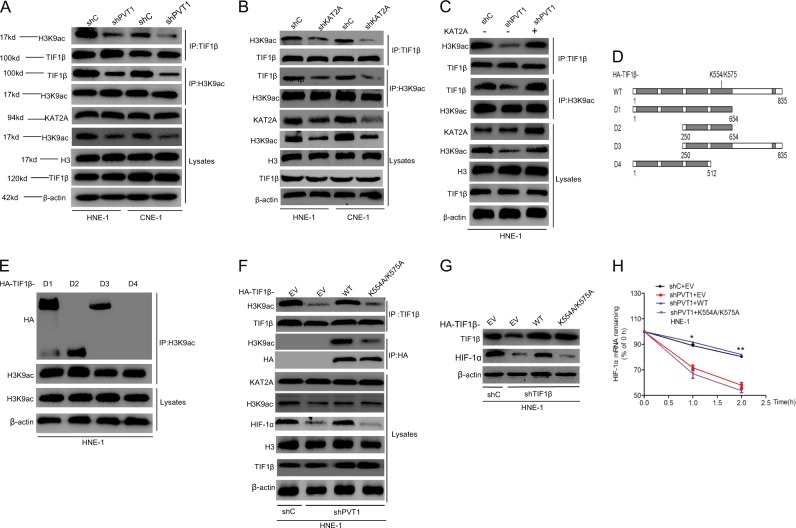
PVT1 promotes HIF-1α stability via KAT2A -mediated regulation of the TIF1β/H3K9ac complex. **a** Immunoprecipitation and WB analyses of effects of PVT1 knockdown on H3K9ac association with TIF1β. **b** KAT2A knockdown inhibited the association of H3K9ac with TIF1β. **c** Overexpression of KAT2A rescues PVT1 knockdown-inhibited the binding of H3K9ac with TIF1β. **d** Schematics of TIF1β^WT^ and various TIF1β deletion mutants. **e** H3K9ac binds with TIF1β with amino acid residues 512–654. **f** Overexpression of TIF1β wild type but not K554A/K575A mutant restores PVT1 knockdown-inhibited the binding of H3K9ac with TIF1β. **g** Overexpression of TIF1β wild type but not K554A/K575A mutant restores TIF1β knockdown-inhibited HIF-1α expression. **h** Overexpression of TIF1β wild type but not K554A/K575A mutant restores PVT1 knockdown-inhibited HIF-1α mRNA stability. Error bars ± SD. *P < 0.05. **P < 0.01. Data are representative from three independent experiments

Recently, the transcription intermediary factor (TIF) family of genes have been reported to bind to H3 acetylation sites in the genome and thereby regulate the expression of proximal genes in many cancers [27–29]. We hypothesized that PVT1 may promote KAT2A-mediated HIF-1α expression via promoting H3K9 acetylation and thereby enhancing the binding of TIF genes and H3K9ac. This would in turn lead TIF family genes to function as transcriptional activators which promote downstream gene transcription, leading to HIF-1α stability and thereby enhancing NPC tumorigenesis. To verify this hypothesis, we performed immunoprecipitation assays in NPC cells. As shown in Supplementary Fig. 5A, we found that H3K9ac bound to TIF1α and TIF1β, but not to TIF1γ, NCOA1, NCOA2, or NCOA3. Next, we determined that overexpression of TIF1β, but not of TIF1α, rescued PVT1 knockdown-mediated loss of HIF-1α mRNA stability (Supplementary Fig. 5B, C). We therefore hypothesized that PVT1 promotes HIF-1α mRNA stability via a TIF1β/H3K9ac complex. We found that knockdown of PVT1 significantly attenuated the binding of TIF1β and H3K9ac, whereas overexpression of KAT2A rescued this defect (Fig. 7a–c and Supplementary 6A–C). Next, to further assess which domain of TIF1β regulates its binding to H3K9ac, we employed a deletion-mapping approach (Fig. 7d), and then transfected these deletion mutant protein isoforms into HNE-1 cells. We found that the D1, D2, and D3 mutants, but not the D4 mutant, were able to bind to H3K9ac (Fig. 7e), suggesting that amino acids 512–654 of TIF1β are required for binding to H3K9ac. To further confirm that TIF1β is important for PVT1-mediated HIF-1α stabilization, we overexpressed either HA-tagged TIF1β WT or a K554A/K575A TIF1β mutant (K554A/K575A) in which the binding of TIF1β and H3K9ac was predicted to be disrupted by The Eukaryotic Linear Motif Resource for Functional Sites of Proteins (http://elm.eu.org). As shown in Fig. 7f and Supplementary Fig.  6D, overexpression of HA-TIF1β WT, but not HA-TIF1β-K554A/K575A mutant, could restored PVT1 depletion-mediated loss of HIF-1α expression instead of H3K9 acetylation. Moreover, overexpression of HA-TIF1β WT was able to rescue PVT1 depletion-mediated loss of binding between TIF1β and H3K9ac, but overexpression of the HA-TIF1β-K554A/K575A mutant did not affect this interaction. Exogenous expression of TIF1β WT could restored TIF1β depletion-mediated loss of HIF-1α expression, but overexpression of the HA-TIF1β-K554A/K575A mutant had no effects (Fig. 7g and Supplementary Fig. 6E). Consistent with these findings, overexpression of HA-TIF1β WT restored PVT1 depletion-mediated loss of cell proliferation, colony formation, and HIF-1α mRNA stability, while overexpression of Flag-TIF1β-K554A/K575A mutant did not alter these characteristics (Fig. 7h, Supplementary Fig. 6F, G). Taken together, these results demonstrate that PVT1 promotes HIF-1α stability through a KAT2A-mediated TIF1β/H3K9ac complex.

### PVT1 promotes TIF1β/H3K9ac complex-mediated NF90 transcriptional activation to regulate HIF-1α stability

**Fig. 8:**
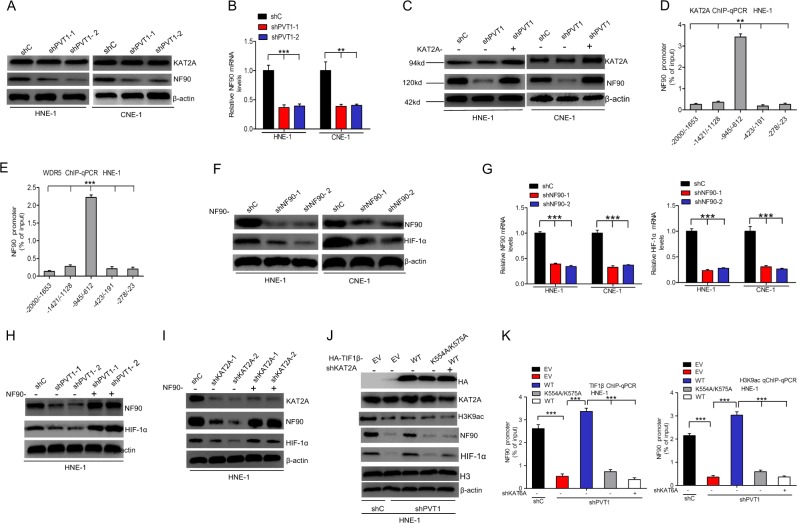
PVT1 promotes TIF1β/H3K9ac complex-mediated NF90 transcriptional activation to regulate HIF-1α stability. Effects of PVT1 knockdown on NF90 protein (**a**) and mRNA (**b**) expression in HNE-1 and CNE-1 NPC cells. **c** Overexpression of KAT2A restored PVT1 knockdown-inhibited NF90 expression. **d**, **e** The ChIP-qPCR assay of the association of KAT2A or WDR5 with the NF90 promoter. After using antibodies against KAT2A, WDR5, or control IgG, qPCR assays were conducted using primers corresponding to five different loci of the NF90 promoter. Effects of NF90 knockdown on HIF-1α protein (**f**) and mRNA (**g**) expression in HNE-1 and CNE-1 NPC cells. Overexpression of NF90 restored PVT1 (**h**) and KAT2A (**i**) knockdown-inhibited NF90 expression. **j** Effects of overexpression of TIF1β wild-type and K554A/K575A mutant on PVT1 knockdown-inhibited HIF-1α, NF90, and the acetylation of H3K9 expression in HNE-1 cells. **k** Representation of overexpression of TIF1β wild type but not K554A/K575A mutant on PVT1 knockdown-inhibited the association of KAT2A or WDR5 with the NF90 promoter. Error bars ± SD. **P < 0.01. ***P < 0.001. Data are representative from three independent experiments

As NF90 is known to stabilize HIF-1α and to regulate its gene expression [30], we further hypothesized that PVT1 may promote HIF-1α mRNA stability via TIF1β/H3K9ac complex-mediated regulation of NF90 transcription. Consistent with this, we found that knockdown of PVT1 suppressed NF90 protein (Fig. 8a and Supplementary Fig. 7A) and mRNA expression levels (Fig. 8b). However, overexpression of KAT2A restored PVT1 depletion-mediated loss of NF90 expression (Fig. 8c and Supplementary Fig. 7B). Furthermore, we performed ChIP assays to determine that KAT2A and WDR5 bind to the NF90 promoter at the −945 to −621 locus (Fig. 8d, e). NF90 knockdown-inhibited HIF-1α protein (Fig. 8f and Supplementary Fig. 7C) and HIF-1α mRNA (Fig. 8g). We further found that overexpression of NF90 restored PVT1 or KAT2A depletion-mediated loss of HIF-1α expression (Fig. 8h–i and Supplementary Fig. 7D, E). Interestingly, overexpression of HA-TIF1β WT rescued PVT1 knockdown-mediated loss of NF90 and HIF-1α, and the binding between the NF90 promoter and the TIF1β/H3K9ac complex (Fig. 8j–k and Supplementary Fig. 7F), while overexpression of HA-TIF1β WT combined with KAT2A knockdown or binding mutant isoforms of the TIF1β/H3K9ac complex alone did not affect it (Fig. 8j–k and Supplementary Fig. 7F). These data thus show that PVT1 promotes TIF1β/H3K9ac complex-mediated NF90 transcription as a means of regulating HIF-1α stability.

### PVT1 suppresses the radiosensitivity of NPC cell lines by increasing the interaction between TIF1β and H3K9ac

**Fig. 9:**
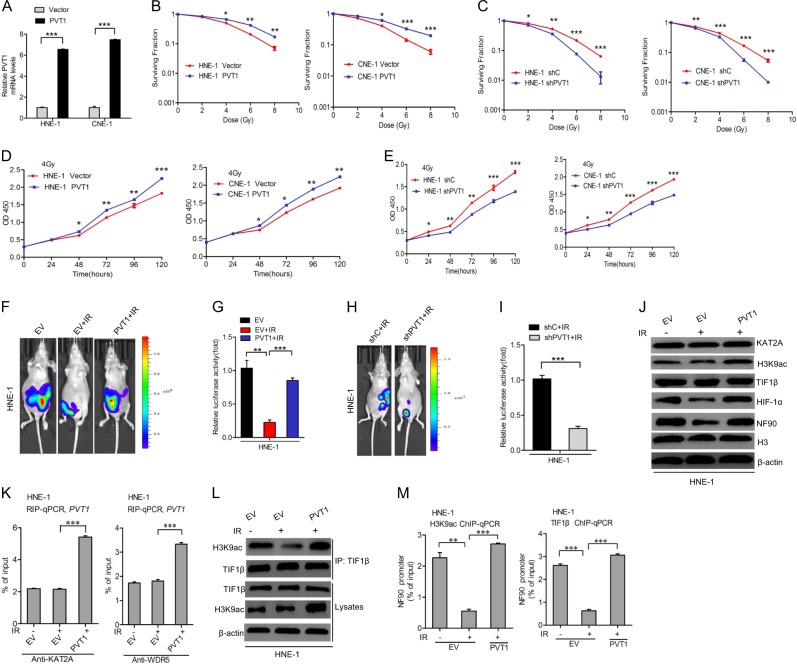
PVT1 suppresses the radiosensitivity of NPC cell lines by increasing the interaction between TIF1β and H3K9ac. **a** Effects of PVT1 overexpression in HNE-1 and CNE-1 NPC cells. Colony formation assays of PVT1 knockdown-(**b**) and overexpression-(**c**) mediated radiosensitivity in HNE-1 and CNE-1 NPC cells. Cell proliferation assays of PVT1 knockdown-(**d**) and overexpression-(**e**) mediated radiosensitivity in HNE-1 and CNE-1 NPC cells. **f** Representative bioluminescence images of PVT1 overexpression-restored radiation-inhibited HNE-1 subcutaneous tumor generation. Mice were imaged at 3–4 weeks after implantation. Data were from two independent experiments with five mice per group with similar results. **g** Quantification of the bioluminescence activity in **f**. **h** Representative bioluminescence images of PVT1 knockdown-induced radiation-inhibited HNE-1 subcutaneous tumor generation. **i** Quantification of the bioluminescence activity in **h**. **j** Overexpression of PVT1 rescues radiation-inhibited HIF-1α and NF90 expression. **k** The RIP assay of radiation has no effect on the association of KAT2A or WDR5, and PVT1. **l** Overexpression of PVT1 rescues radiation-inhibited the interaction between TIF1β and H3K9ac. **m** Effects of PVT1 overexpression on radiation-inhibited the association of KAT2A or WDR5 with the NF90 promoter. Error bars ± SD. *P < 0.05. **P < 0.01. ***P < 0.001. Data are representative from three independent experiments

As radiation is a primary treatment for NPC, and PVT1 suppresses radiosensitivity in NPC [20], we reasoned that the molecular mechanism by which PVT1 regulates cancer progression may be closely related to NPC radiosensitivity. We found that PVT1 overexpression significantly increased the number of colonies formed when cells were treated via radiation relative to untreated controls in a dose-dependent manner (Fig. 9a, b), whereas PVT1 knockdown showed the opposite effects (Fig. 9c). Importantly, we found that PVT1 overexpression significantly increased cell proliferation and PVT1 knockdown markedly decreased cell proliferation after exposure to 4 Gy radiation (Fig. 9d, e). Consistent with this, tumor growth in vivo also recapitulated these findings (Fig. 9f–i).

To investigate how PVT1 suppresses cell proliferation after radiation, we first examined that radiation had no effects on KAT2A, H3K9ac, or TIF1β protein expression, but did inhibit NF90 and HIF-1α expression (Fig. 9j and Supplementary Fig. 8A). However, overexpression of PVT1 restored radiation-mediated loss of NF90 and HIF-1α expression (Fig. 9j and Supplementary Fig. 8A). Furthermore, radiation did not affect the binding of PVT1, KAT2A, and WDR5 (Fig. 9k). We thus hypothesized that radiation disturbed the interaction between H3K9ac and TIF1β, thereby decreasing NF90 transcription. To test our hypothesis, we detected that radiation inhibited the interaction between H3K9ac and TIF1β, and thus suppressed H3K9ac and TIF1β-mediated enhancement of NF90 transcription (Fig. 9l, m and Supplementary Fig. 8B). In conclusion, these results show that PVT1 suppresses the radiosensitivity of NPC cell lines by disturbing the interaction between TIF1β and H3K9ac.

## Discussion

**Fig. 10:**
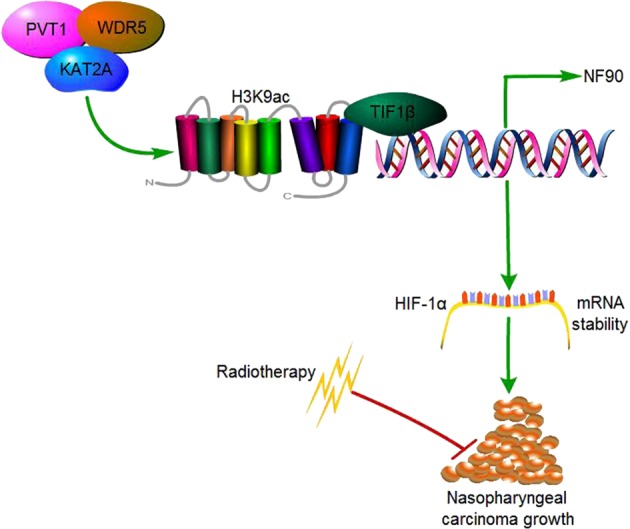
A working model for PVT1-regulated NPC tumorigenesis. PVT1 acts as a scaffold of KAT2A and WDR5, and KAT2A promotes H3K9 acetylation and association with TIF1β, leading to increased NF90 transcription and HIF-1α stabilization, thus resulting in enhanced NPC tumorigenesis

In this study, we identify a new putative mechanism by which the lncRNA PVT1 serves as a scaffold for the chromatin modification factor KAT2A, and thereby stabilizes HIF-1α via H3K9ac/TIF1β complex-mediated NF90 transcription in a manner critical for NPC tumorigenesis (Fig. 10).

Our results reveal that PVT1 functions as an oncogene in the context of NPC tumorigenesis. Increasing evidences demonstrate that abnormal expression of many lncRNAs can play a critical role in malignant NPC progression [20, 30]. Recent PVT1 was found to promote cell proliferation via regulating the cell cycle in many cancers [31, 32]. In this study, we found that expression of PVT1 was upregulated in NPC specimens. Moreover, PVT1 expression is negatively correlated with survival in NPC patients. PVT1 depletion via shRNA inhibited NPC cell proliferation and colony formation in vitro, and NPC tumorigenesis in vivo. These results therefore support a model in which PVT1 is critical for NPC tumorigenesis.

Our results also reveal that PVT1 mediates NPC cell proliferation via HIF-1α stabilization. Although PVT1 contributed to the regulation of cell proliferation in cancer cells [31, 32], the mechanisms by which PVT1 regulates cell proliferation in NPC remained unclear. PVT1 knockdown predicts poor prognostic and induces radioresistance by mediate cell apoptosis and DNA repair in NPC [20]. PVT1 promotes colony formation via the PI3K/AKT signaling pathway in NPC [33]. Our KGEE analysis demonstrated that PVT1 expression was positively correlated with a hypoxia phenotype. This is consistent with recent clinical data showing that primary nasopharynx tumors are primarily hypoxic [34, 35], suggesting that a hypoxic environment may be crucial for NPC progression. Moreover, HIF-1α, a core mediator of the hypoxia response [36, 37], was downregulated after PVT1 depletion. In addition, HIF-1α mRNA stability has also been reported to be regulated by lncRNAs and then affects NPC carcinogenesis [30, 38]. Our results also showed that the knockdown of PVT1 reduced HIF-1α mRNA stability, which was restored by overexpression of HIF-1α. NF90, known as a double-stranded RNA-binding protein, is transcribed from the interleukin enhancer binding factor 3 gene. A growing body of evidence show that NF90 regulates mRNA stability and plays a vital role in RNA metabolism [39, 40]. Thus, we hypothesized that PVT1 might increase HIF-1α mRNA stability via activating NF90 transcription. Indeed, the ChIP assay confirmed that PVT1 knockdown suppressed NF90 transcription and NF90 overexpression rescued PVT1 depletion-inhibited HIF-1α mRNA stability. Taken together, our data show that PVT1 regulates NPC cell proliferation through HIF-1α stabilization.

Our results further revealed that PVT1 serves as a scaffold for the chromatin modification factor KAT2A, which stabilizes HIF-1α through H3K9ac/TIF1β complex-mediated NF90 transcriptional activation. Previous work has shown that the lncRNAs GClnc1 and GCAWKR can serve as scaffolds for KAT2A and WDR5 in gastric cancer, and can thereby serve as positive regulators of this enzyme [5, 41]. The lncRNA NEAT1 also interacts with KAT2A to promote deterioration of hepatocellular carcinoma [6]. However, the relationship of PVT1 and KAT2A has not previously been reported. Here, we found that PVT1 can interact with KAT2A, and thereby promotes enhanced H3K9 acetylation in NPC cells. KAT2A-mediated H3K9 acetylation in turn induces the binding of TIF1β to chromatin. Consistent with previous studies of HIF-1α mRNA stability [30], our data demonstrate that overexpression of KAT2A or TIF1β upregulate PVT1-mediated NF90 transcriptional activation in NPC cells in order to increase HIF-1α mRNA stability, while overexpression of KAT2A acetyltransferase activity-deficient mutants or TIF1β mutants lacking H3K9ac binding sites does not. Our results thus demonstrate that PVT1 serves as a scaffold for KAT2A-mediated regulation of HIF-1α mRNA stability in a manner dependent on its acetyltransferase activity and on H3K9ac/TIF1β complex-mediated NF90 transcriptional activation.

Radiation is a primary treatment for NPC [20]. PVT1-mediated suppression of tumor radiosensitivity has been reported in non-small cell lung cancer [42], and NPC [20], respectively. However, the specific underlying signaling pathways were not well characterized. Our data suggest that radiation inhibits NPC cell proliferation via abrogating the binding between H3K9ac and TIF1β, and that this binding is restored by overexpression of PVT1, whereas the binding between PVT1 and KAT2A is unaffected.

In summary, our data identify PVT1 as a potential treatment target in individuals with NPC. Our results further demonstrate a mechanism whereby PVT1 promotes NPC cell proliferation via a KAT2A/H3K9ac/TIF1β/NF90/HIF-1α signaling pathway. Utilization of these results clinically may allow for better personalized radiation dosing based on PVT1 expression in NPC patients.

## Materials and methods

### Clinical specimens

Ten freshly frozen normal nasopharyngeal samples and ten NPC biopsy samples were collected from Zhongshan City People’s Hospital. In addition, 100 formalin-fixed paraffin-embedded NPC tissues were also obtained from Zhongshan City People’s Hospital. between January 2010 and December 2015. All patients had not received radiotherapy and chemotherapy before biopsy. Written patient consent and approval from the Institutional Ethical Review Boards of the Meizhou People’s Hospital were obtained. The usage of specimens were carried out according to the approved guidelines of the Zhongshan City People’s Hospital.

### Cell culture

Human NPC cell lines (SUNE-1, C666-1,CNE-2, CNE-1, and HNE-1) were obtained from Cell Bank of the Chinese Scientific Academy (Shanghai, China), and were cultured in Dulbecco’s modified Eagle’s medium (Invitrogen, Carlsbad, CA) supplemented with 10% fetal bovine serum. NP69 was also purchased from Cell Bank of the Chinese Academy of Sciences (Shanghai, China), and was cultured in DMEM/F12 (Invitrogen, Carlsbad, CA) supplemented with 5% horse serum, 20 ng/ml epidermal growth factor, 0.5 μg/ml hydrocortisone, 100 ng/ml cholera toxin, 10 μg/ml insulin, and 100 μg/ml penicillin–streptomycin. Cells were cultured in a humidified incubator in a 5% CO_2_ atmosphere at 37 °C. Hypoxic conditions were achieved with a hypoxia chamber (Billups-Rothenberg) flushed with a gas mixture of 1% O_2_, 5% CO_2_ and 94% N_2_.

### RNA extraction, reverse transcription, and quantitative PCR

TRIzol reagent (Invitrogen) was utilized to isolate total RNA from NCP tissues and cells. The RNeasy FFPE kit (QIAGEN GmbH) was utilized to extract total RNA from FFPE NPC tissues. The RNA quality and amount were evaluated by a NanoDrop 3300 spectrophotometer (Thermo Scientific). Reverse transcriptase (Promega) was utilized to perform reverse transcription. SYBR Green qPCR SuperMix-UDG (Thermo Fisher) was utilized to conduct Quantitative real-time PCR. β-actin was used as the normalization control. Specific primers are shown in Supplenentary Table S1.

### Western blotting

Western blot was conducted as we previously described [43]. The antibodies for western blotting are listed as follows: β-actin (#4970, 1:1000, Cell Signaling Technology), KAT2A (#3305S, 1:1000, Cell Signaling Technology), HIF-1α (#36169, 1:1000, Cell Signaling Technology), H3K9ac (#9649, 1:1000, Cell Signaling Technology), Histone H3 (#4499, 1:1000, Cell Signaling Technology), TIF1γ (#13387, 1:1000, Cell Signaling Technology), TIF1β (#4124, 1:1000, Cell Signaling Technology), TIF1α (ab38264, 1:1000, Abcam), NCOA1 (#20301, 1:1000, Cell Signaling Technology), NCOA2 (#96687, 1:1000, Cell Signaling Technology), NCOA3 (#2126, 1:1000, Cell Signaling Technology), NF90 (sc-377406, 1:500, Santa Cruz Biotechnology), WDR5 (#13105,1:1000, Cell Signaling Technology), KAT2B (#3378, 1:1000, Cell Signaling Technology), KAT5 (#12058,1:1000, Cell Signaling Technology), KAT8 (#46862, 1:1000, Cell Signaling Technology), and KAT6A (ABP59003, 1:1000, Abbkine).

### ChIP-qPCR

The chromatin immunoprecipitation kit (Millipore-17-408) was utilized to perform the ChIP assay according to the manufacturer’s instruction. The immunoprecipitated DNAs were quantified through quantitative real-time PCR. Specific primers are shown in Supplenentary Table S1.

### In situ hybridization

The in situ detection of PVT1 was performed on paraffin-embedded sections using the RNAscope 2.5 HD Detection Reagent-BROWN kit (ACDBio). The specific PVT1 probes were made by ACDBio.

### Construction of vectors

The cDNA encoding PVT1, KAT2A, TIF1β, and NF90 were amplified from NP69 cells and sequenced, and then subcloned into the pcDNA3 vector (Invitrogen), subsequently named pCDNA3-PVT1, pCDNA3-KAT2A, pCDNA3-TIF1β, and pCDNA3-NF90. pLVX-PVT1, pLVX-KAT2A, pLVX-TIF1β, and pLVX-NF90 was generated from pCDNA3-PVT1, pCDNA3-KAT2A, pCDNA3-TIF1β, and pCDNA3-NF90, respectively. NF90 promoter was PCR-amplified from NP69 cells and sequenced, and then subcloned into pGL3 vector (Promega). A Quik Change Site-Directed Mutagenesis Kit (Stratagene) was used for point mutations. shRNAs were designed as follows: PVT1 (shPVT1-1 target sequence: 5′-GCCATCATGATGGTACTTTAA-3′; shPVT1-2 target sequence 5′-GCCA GGACACTG AGATTTGGA-3′); KAT2A (shKAT2A-1 target sequence: 5′-CGTGCTGTCACCTCGAATGA-3′; shKAT2A-2 target sequence 5′-TCATGTCTGTTCACAAGGAA-3′); TIF1β (shTIF1β target sequence: 5′-TCTGTTCTCTGTCCTCTCGAGAGGACAGAGAACAGAGCCAGG-3′); NF90 (shNF90-1 target sequence: 5′-GTGCTGGTTCCAACAAAA-3′; shNF90-2 target sequence 5′-AGTCGT GGAAAGCCTAAGA-3′). shRNAs was cloned into lentiviral expression vector pLL3.7.

### Colony formation and CCK8 assays

For the colony formation assay, 600 cells in 2 ml medium were seeded into six-well plates and cultured for 6 or 11 days. Cell colonies were then successively fixed, stained, and counted. For the CCK8 assay, 2 × 10^3^ cells in 100 μl medium solution were cultured in 96-well plates. Then, a 10 μl CCK8 reagent (DOJINDO) was added to each well. After the 96-well plates were incubated for 2 h at 37 °C, we then measure the absorbance at 450 nm for each sample well.

### RNA immunoprecipitation (RIP) and RNA pull-down assays

RIP and RNA pull-down assays were performed as we previously described [44]. Antibodies used for RIP are displayed as follows: WDR5 (#13105,1:50, Cell Signaling Technology), KAT2B (#3378, 1:50, Cell Signaling Technology), KAT5 (#12058,1:50, Cell Signaling Technology), KAT8 (#46862, 1:50, Cell Signaling Technology), KAT6A (ABP59003, 1:100, Abbkine), and KAT2A (#3305S, 1:50, Cell Signaling Technology).

### RNA sequencing

Total RNA was extracted from HNE-1 transfected with shPVT1 or shC control and three free-frozen NPC tissues and corresponding normal nasopharyngeal specimens. The RNA quality was measured through Agilent Bioanalyzer 2100 (Agilent technologies). Qualified RNA was then purified by RNA Clean XP Kit (A63987, Beckman) and Qiagen RNase-Free DNase Set (79254, QIAGEN).

Next, we constructed Illumina sequencing libraries according to the instruction. Briefly, we extracted noncoding and coding RNA from 5 μg of purified total RNA using a Ribo-Zero rRNA Removal Kit. After isolation, we then eluted and fragmented the RNA samples at 94 °C for 8 min with Elute–Prime–Fragment mix. Subsequently, we synthesized the first-strand cDNA using Invitrogen SuperScript II. Then, Second-Strand Marking Master Mix mixed with the product was incubated at 16 °C for 1 h to synthesize the second-strand cDNA. After 3′ ends adenylation, we ligated the DNA fragments with TruSeq adapters and the product was amplified using TruSeq PCR primers. Then, the libraries were loaded on cBot (Illumina) to generate a cluster and subsequently sequenced on the HiSeq X ten (Illumina) platform at the Ling En biotechnological Corporation (Shang hai).

RNA-seq reads were mapped to the human reference genome sequence (hg38) assembly by Hisat2 (Version 2.1.0). Stringtie (version 1.3.3) was used to count the number of fragments of each known gene downloaded from Ensembl (Homo sapiens GRCh38). After mapping, we removed genes with fewer than ten fragments in all of the samples and identified differentially expressed genes using R/Bioconductor package edge R (version 3.0.8). Thresholds of *P* < 0.05 and fold change ≥ 1.5 were used to identify differentially expressed genes.

The differentially expressed genes were used to perform KEGG (Kyoto Encyclopedia of Genes and Genomes) pathway analysis and GO analysis using DAVID software (https://david.ncifcrf.gov/). Two-sided or Bonferroni-corrected Fisher’s exact tests were used to calculate the *P*-values of KEGG pathways or GO terms, respectively. A threshold of *P* < 0.05 and FDR ≤ 0.25 was used to select significant items. RNA data are available at the NCBI BioProject database (http://www.ncbi.nlm.nih.gov/bioproject) under Bioproject ID: PRJNA548225.

### Tumorigenesis studies

Four- to five-week-old female BALB/c nude mice (SLAC) were randomly divided into per group, and then HNE-1 cells (2 × 10^6^) were implanted subcutaneously into each mice. All animal experiments procedures were performed according to guidelines approved by Zhejiang Provincial People’s Hospital the Guidance of Institutional Animal Care and Use Committee. As previously described [17], HNE-1 cells were transduced with a luciferase reporter plasmids. Tumor volumes were measured using the largest tumor cross section for each samples in vivo using luciferase activity after injection of d-luciferin. The IVIS Lumina imaging station (Caliper Life Sciences) was used for bioluminescence imaging. Different investigators independently performed mice allocation, surgery and the outcome assessing.

### Immunohisochemical staining of human NPC specimens

Human NPC specimens including paraffin-embeddeded-identified NPC tissues and corresponding normal nasopharyngeal specimens were obtained from Zhongshan City People’s Hospital. The specimens were stained with antibodies against KAT2A (1:50) and WDR5 (1:50). Immunohisochemical staining was scored as previously described [40].

### Statistical analysis

The data were conducted using the GraphPad Prism version 5.0. The cumulative survival rate was evaluated using the Kaplan–Meier method. The statistical analyses of data from experimental groups was determined by multiple comparison tests. *P < 0.05 was considered statistically significant.

## Supplementary information

Supplementary Figure 1

Supplementary Figure 2

Supplementary Figure 3

Supplementary Figure 4

Supplementary Figure 5

Supplementary Figure 6

Supplementary Figure 7

Supplementary Figure 8

Supplementary Table 1
