# Supplementary material for: The lncRNA PVT1 regulates nasopharyngeal carcinoma cell proliferation via activating the KAT2A acetyltransferase and stabilizing HIF-1α
Source: Cell Death Differ. 2019 Jul 18;27(2):695–710. doi: 10.1038/s41418-019-0381-y (PMC7206084; doi:10.1038/s41418-019-0381-y)
Supplement: Supplementary file 1 — Supplementary Figure 1 [file 41418_2019_381_MOESM1_ESM.pdf]

A

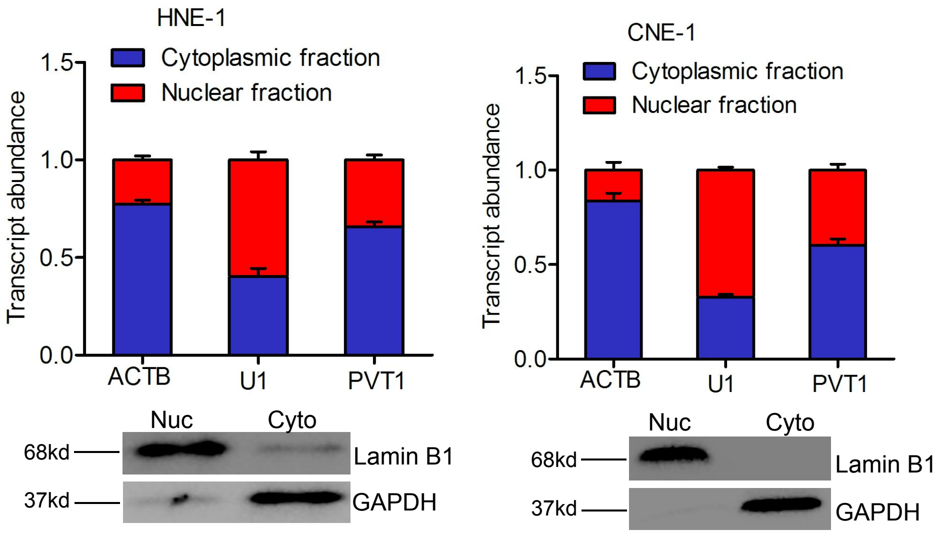

B

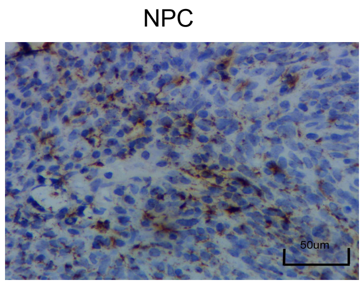

Supplementary Figure 1. **PVT1 localization in NPC cells. A**, Real-time PCR analysis of the subcellular location of PVT1 in NPC cells. U1 snRNA (nuclear retained) and ACTB mRNAs (exported to cytoplasm) were used as controls. **B**, Representative images of PVT1 expression in clinical NPC tissues using RNAscope analysis.
