# Supplementary material for: The lncRNA PVT1 regulates nasopharyngeal carcinoma cell proliferation via activating the KAT2A acetyltransferase and stabilizing HIF-1α
Source: Cell Death Differ. 2019 Jul 18;27(2):695–710. doi: 10.1038/s41418-019-0381-y (PMC7206084; doi:10.1038/s41418-019-0381-y)
Supplement: Supplementary file 2 — Supplementary Figure 2 [file 41418_2019_381_MOESM2_ESM.pdf]

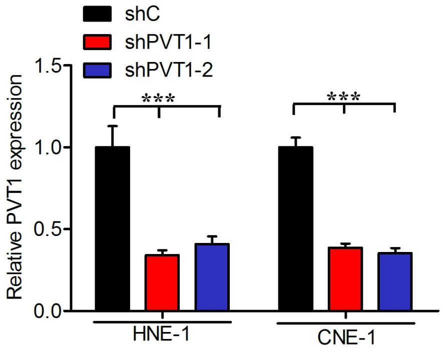

Supplementary Figure 2. **Effects of shRNA knockdown on PVT1 mRNA expression in HNE-1 and CNE-1 NPC cells**. Error bars ±SD. ***P <0.001. Data are representative from three independent experiments.
