# Supplementary material for: The lncRNA PVT1 regulates nasopharyngeal carcinoma cell proliferation via activating the KAT2A acetyltransferase and stabilizing HIF-1α
Source: Cell Death Differ. 2019 Jul 18;27(2):695–710. doi: 10.1038/s41418-019-0381-y (PMC7206084; doi:10.1038/s41418-019-0381-y)
Supplement: Supplementary file 3 — Supplementary Figure 3 [file 41418_2019_381_MOESM3_ESM.pdf]

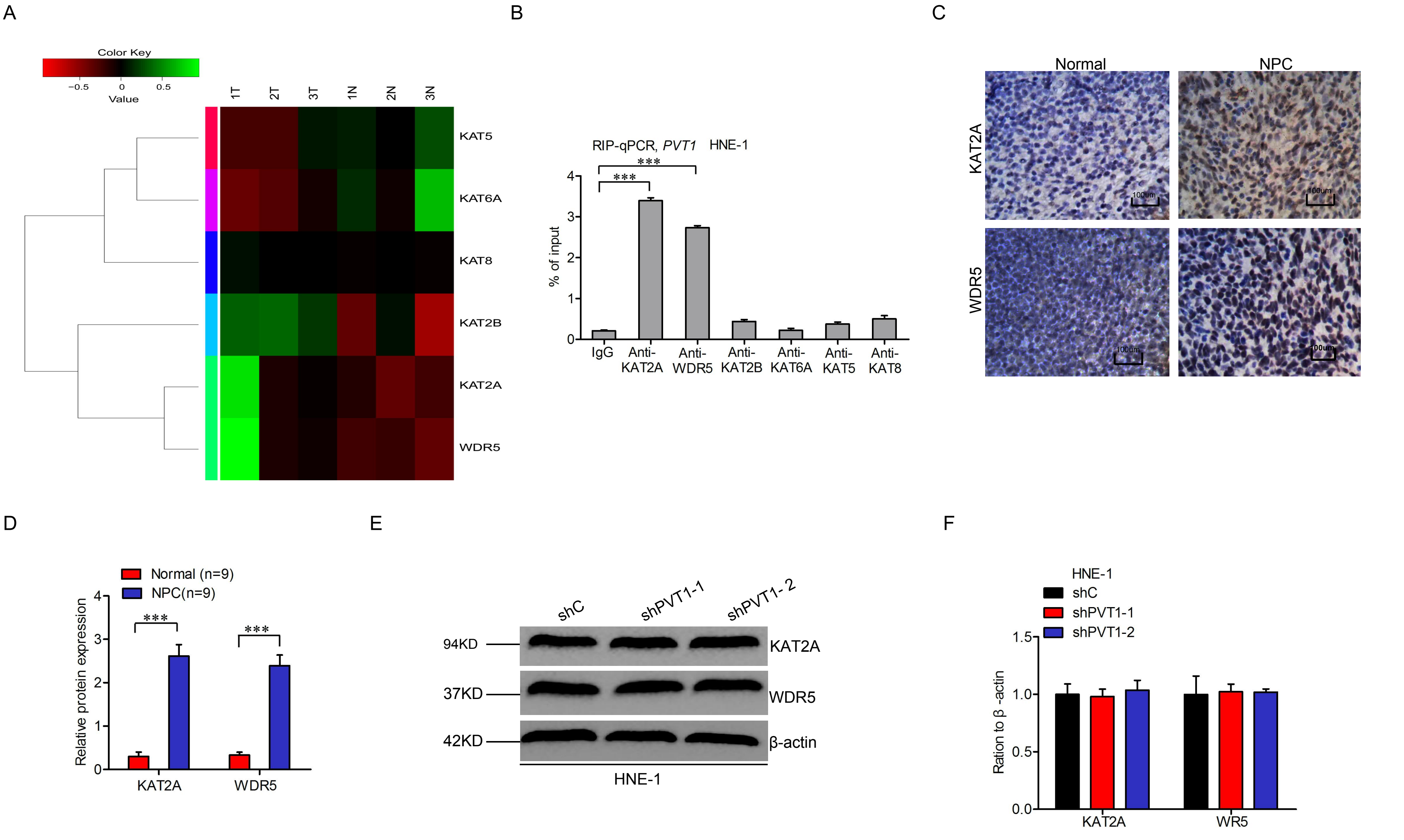

Supplementary Figure 3. PVT1 binds with KAT2A and WDR5 in NPC cells. A,Heatmap illustrating the 6 differentially expressed genes between 3 freshly-frozen normal nasopharyngeal specimens and 3 clinical tumors of NPC patients (fold change ≥ 1.5, P-value < 0.05). **B**, Effects of the association of KAT2A, WDR5, KAT2B, KAT6A, KAT5 or KAT8 ,and PVT1. **C**, Immunohistochemistry staining assay of KAT2A and WDR5 in 9 normal nasopharyngeal specimens and 9 clinical NPC tumors. **D**, Quantification analysis of KAT2A and WDR5 in **C**. **E**, Effects of PVT1 knockdown on KAT2A and WDR5 expression. **F**.Quantification analysis of KAT2A and WDR5 in **E**. Error bars ±SD. ***P <0.001. Data are representative from three independent experiments.
