# Supplementary material for: The lncRNA PVT1 regulates nasopharyngeal carcinoma cell proliferation via activating the KAT2A acetyltransferase and stabilizing HIF-1α
Source: Cell Death Differ. 2019 Jul 18;27(2):695–710. doi: 10.1038/s41418-019-0381-y (PMC7206084; doi:10.1038/s41418-019-0381-y)
Supplement: Supplementary file 4 — Supplementary Figure 4 [file 41418_2019_381_MOESM4_ESM.pdf]

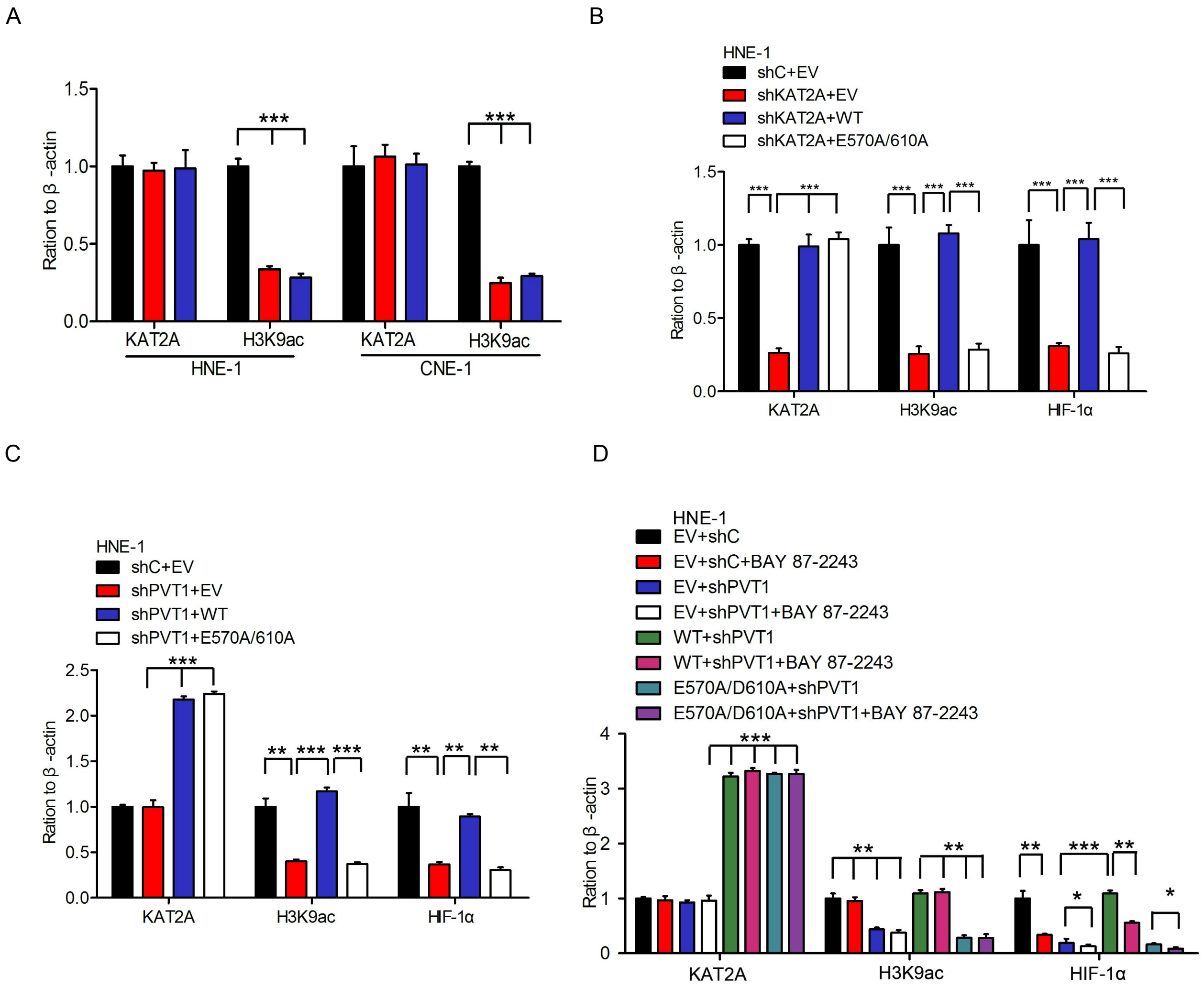

Supplementary Figure 4. **A**.Quantification analysis of KAT2A and H3K9ac in **Figure6A. B**. Quantification analysis of KAT2A, H3K9ac and HIF-1α in **Figure6B. C**. Quantification analysis of KAT2A, H3K9ac and HIF-1α in **Figure6C**. **D**.Quantification analysis of KAT2A, H3K9ac and HIF-1α in **Figure6F**. Error bars ±SD. **P<0.01. ***P <0.001. Data are representative from three independent experiments.
