# Supplementary material for: The lncRNA PVT1 regulates nasopharyngeal carcinoma cell proliferation via activating the KAT2A acetyltransferase and stabilizing HIF-1α
Source: Cell Death Differ. 2019 Jul 18;27(2):695–710. doi: 10.1038/s41418-019-0381-y (PMC7206084; doi:10.1038/s41418-019-0381-y)
Supplement: Supplementary file 5 — Supplementary Figure 5 [file 41418_2019_381_MOESM5_ESM.pdf]

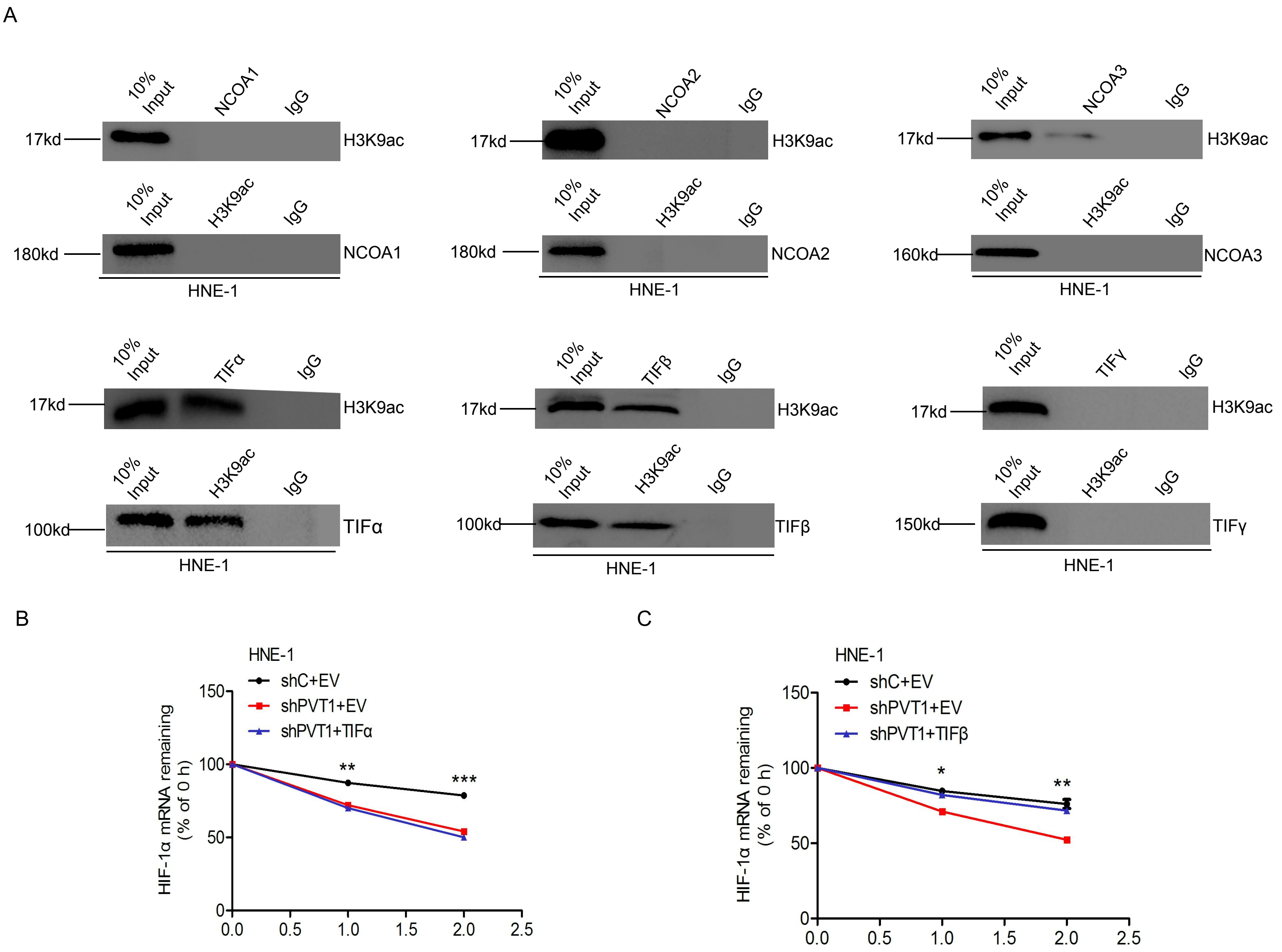

Supplementary Figure 5. PVT1 regulates HIF-1α through binding TIF1β. A, Immunoprecipitation and WB analyses of effects of H3K9ac association with NCOA1, NCOA2, NCOA3, TIF1α, TIF1β, and TIF1γ. **B-C**, TIF1β but not TIF1α rescues PVT1 knockdown-inhibited HIF-1α mRNA stability. Error bars ±SD. *P<0.05. **P<0.01. ***P <0.001. Data are representativefrom three independent experiments.
