# Supplementary material for: The lncRNA PVT1 regulates nasopharyngeal carcinoma cell proliferation via activating the KAT2A acetyltransferase and stabilizing HIF-1α
Source: Cell Death Differ. 2019 Jul 18;27(2):695–710. doi: 10.1038/s41418-019-0381-y (PMC7206084; doi:10.1038/s41418-019-0381-y)
Supplement: Supplementary file 6 — Supplementary Figure 6 [file 41418_2019_381_MOESM6_ESM.pdf]

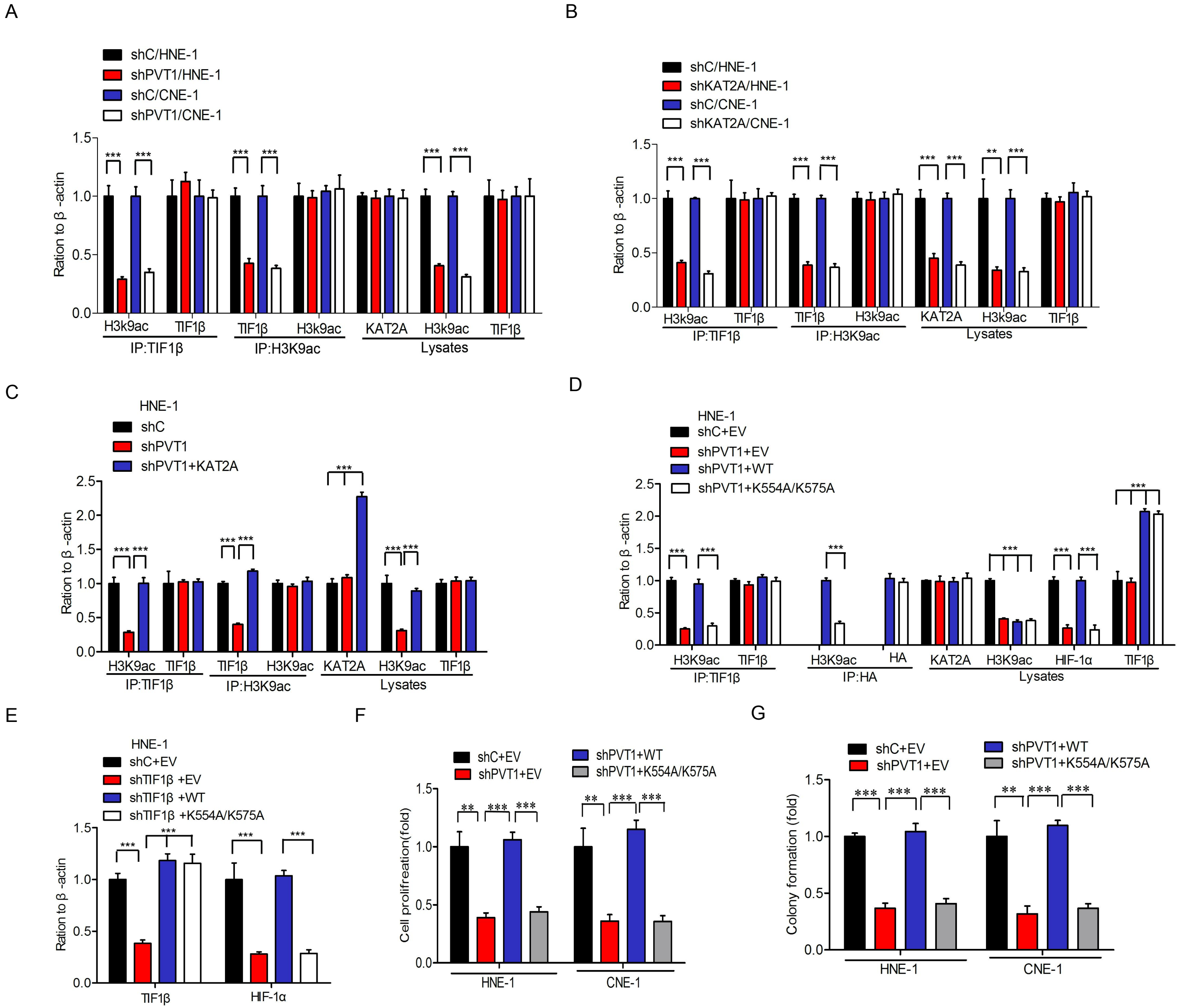

Supplementary Figure 6. **A**.Quantification analysis of **Figure7A. B**. Quantification analysis of **Figure7B. C**. Quantification analysis of **Figure7C**. **D**. Quantification analysis of **Figure7F**. **E**. Quantification analysis of **Figure7G**. Representation of overexpression of TIF1β wild type but not K554A/K575A mutant on PVT1knockdown-inhibited cell proliferation (**F**) and colony formation (**G**). Error bars ±SD. **P<0.01. ***P <0.001. Data are representative from three independent experiments.
