# Supplementary material for: The lncRNA PVT1 regulates nasopharyngeal carcinoma cell proliferation via activating the KAT2A acetyltransferase and stabilizing HIF-1α
Source: Cell Death Differ. 2019 Jul 18;27(2):695–710. doi: 10.1038/s41418-019-0381-y (PMC7206084; doi:10.1038/s41418-019-0381-y)
Supplement: Supplementary file 7 — Supplementary Figure 7 [file 41418_2019_381_MOESM7_ESM.pdf]

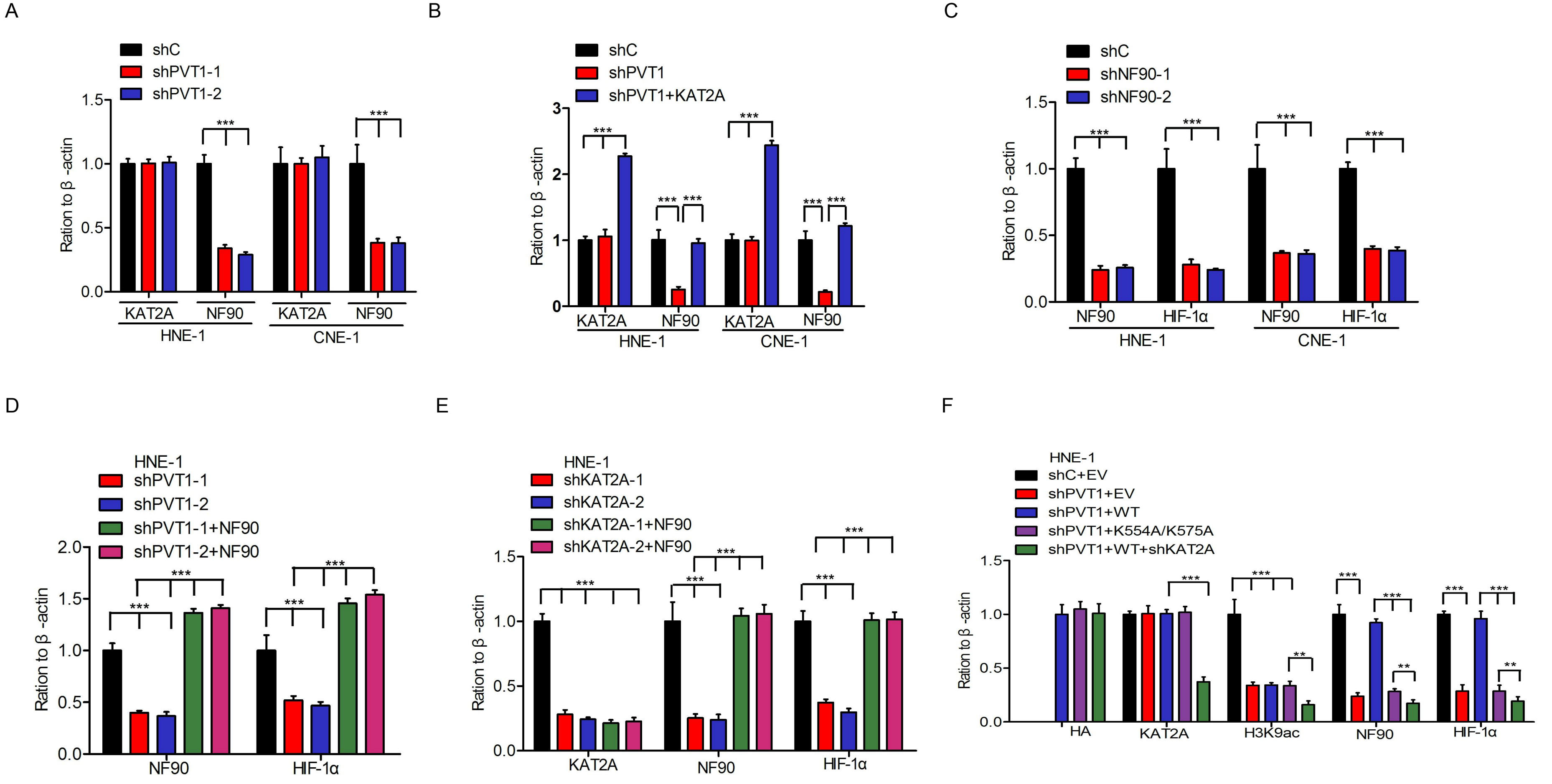

Supplementary Figure 7. **A**.Quantification analysis of **Figure8A. B**. Quantification analysis of **Figure8C. C**. Quantification analysis of **Figure8F**. **D**. Quantification analysis of **Figure8H**. **E**. Quantification analysis of **Figure8I**. **F**. Quantification analysis of **Figure8J.** Error bars ±SD. ***P <0.001. Data are representative from three independent experiments.
