# Supplementary material for: The lncRNA PVT1 regulates nasopharyngeal carcinoma cell proliferation via activating the KAT2A acetyltransferase and stabilizing HIF-1α
Source: Cell Death Differ. 2019 Jul 18;27(2):695–710. doi: 10.1038/s41418-019-0381-y (PMC7206084; doi:10.1038/s41418-019-0381-y)
Supplement: Supplementary file 9 — Supplementary Table 1 [file 41418_2019_381_MOESM9_ESM.docx]

**Supplementary Table 1** Primers for qRT-PCR assays and ChIP-qPCR assays

| Primer pairs | Sequence |
| --- | --- |
| qRT-PCR,β-actin | 5’-CATGTACGTTGCTATCCAGGC-3’ and  5’-CTCCTTAATGTCACGCACGAT-3’ |
| qRT-PCR,PVT1 | 5’-TGAGAACTGTCCTTACGTGACC-3’ and  5’-AGAGCACCAAGACTGGCTCT-3’ |
| qRT-PCR,HIF-1α | 5’-GAACGTCGAAAAGAAAAGTCTCG-3’ and  5’-CCTTATCAAGATGCGAACTCACA-3’ |
| qRT-PCR,NF90 | 5’-AGCATTCTTCCGTTTATCCAACA-3’ and  5’-GCTCGTCTATCCAGTCGGAC-3’ |
| PVT1 shRNA #1 | 5’-TGCCATCATGATGGTACTTTAACGAATTAAAGTACCATCATGATGGCTTTTC-3’and  5’-TCGAGAAAAGCCATCATGATGGTACTTTAATTCGTTAAAGTACCATCATGATGGCA-3’ |
| PVT1 shRNA #2 | 5’-TGCCAGGACACTGAGATTTGGACGAATCCAAATCTCAGTGTCCTGGCTTTTC**-**3’ and  5’-TCGAGAAAAGCCAGGACACTGAGATTTGGATTCGTCCAAATCTCAGTGTCCTGGCA-3’ |
| KAT2A shRNA #1 | 5’-TGGCTCCCCGGGAGCGGCAGACCGAAGTCTGCCGCTCCCGGGGAGCCTTTTC-3’and  5’-TCGAGAAAAGGCTCCCCGGGAGCGGCAGACTTCGGTCTGCCGCTCCCGGGGAGCCA-3’ |
| KAT2A shRNA #2 | 5’-TGAGCGTTCCTGGCATTCGAGACGAATCTCGAATGCCAGGAACGCTCTTTTC-3’and  5’-TCGAGAAAAGAGCGTTCCTGGCATTCGAGATTCGTCTCGAATGCCAGGAACGCTCA-3’ |
| ChIP-qPCR,NF90  -2000 to -1653 | 5’-TGAAGTAAGCTGGCTAA-3’ and  5’-GATTGCTCCATTGCACT-3’ |
| ChIP-qPCR,NF90  -1421 to -1128 | 5’-CCAGGAAACCCATTTTC-3’ and  5’-GCACTTTGGGAGGCCGAG-3’ |
| ChIP-qPCR,NF90  -945 to -612 | 5’-CTCATAAGGCGCCTGGA-3’ and  5’-GCGCCAATGCGCGCCCGG-3’ |
| ChIP-qPCR,NF90  -423 to -191 | 5’-CGGAGCCATTTCCGCTAAC-3’ and  5’-ATTTCGCGGTTTCCTTC-3’ |
| ChIP-qPCR,NF90  -278 to -23 | 5’-GCGTGCCCTCGTCACG-3’ and  5’-AGGCCCAACCCAAATG-3’ |
